# Comparative Evaluation of Fracture Resistance in Endodontically Treated Posterior Teeth Restored With Endocrowns Versus Conventional Post and Core Followed by a Full Crown: An In Vitro Study

**DOI:** 10.7759/cureus.105868

**Published:** 2026-03-26

**Authors:** Mansi Manohar Gadhe, Nida Mehmood, Mridusmita Mukherjee, Karishma Pathak, Ubaid Ullah Gagroo, Aniket Mone, Seema Gupta

**Affiliations:** 1 Department of Prosthodontics and Crown and Bridge, Government Dental College and Hospital, Srinagar, IND; 2 Department of Conservative Dentistry and Endodontics, Kothiwal Dental College & Research Centre, Moradabad, IND; 3 Department of Conservative Dentistry and Endodontics, Government Dental College, Silchar, IND; 4 Department of Conservative Dentistry and Endodontics, Government Dental College, Dibrugarh, IND; 5 Department of Prosthodontics and Crown and Bridge, Vidarbha Youth Welfare Society's Dental College and Hospital, Amravati, IND; 6 Department of Orthodontics, Kothiwal Dental College & Research Centre, Moradabad, IND

**Keywords:** endocrowns, failure, fracture resistance, post and core, stress

## Abstract

Introduction

Endodontically treated posterior teeth are weakened by the loss of coronal structure and require durable restoration to prevent fracture. Conventional post-core-crown systems often involve additional dentin removal, which increases the risk of root fracture. Endocrowns may offer a conservative alternative that uses the pulp chamber for retention. This in vitro study aimed to compare the fracture resistance and failure modes of endodontically treated mandibular first molars restored with lithium disilicate endocrowns versus fiber post-core and lithium disilicate full crowns.

Materials and methods

Sixty extracted mandibular first molars were root canal treated, randomly divided into two groups (n = 30 each), and restored: Group 1 with computer-aided design and computer-aided manufacturing lithium disilicate endocrowns and Group 2 with fiber posts, composite cores, and lithium disilicate crowns. All specimens were cemented with dual-cure resin cement, stored in water for 24 hours, embedded in acrylic, and tested under compressive load (1 mm/min) using a universal testing machine. The fracture load (N) and failure mode (favorable versus unfavorable) were recorded. Data were analyzed using the Shapiro-Wilk test, independent samples t-test, and descriptive statistics (α = 0.05).

Results

The mean fracture resistance of teeth restored with lithium disilicate endocrowns was 1250.45 ± 152.30 N, whereas the post-core-crown group demonstrated a mean value of 1180.20 ± 178.60 N. Although the endocrown group exhibited slightly higher fracture resistance, the difference between the groups was not statistically significant (p = 0.106). Failure mode analysis revealed a marked difference in the pattern of fractures between the groups. Endocrowns showed a significantly higher proportion of favorable (restorable) failures, primarily involving debonding or fractures above the cemento-enamel junction. In contrast, the post-core-crown group exhibited a higher incidence of unfavorable (non-restorable) fractures, including vertical root fractures and post-related failures.

Conclusions

Within the limitations of this in vitro study, lithium disilicate endocrowns demonstrated fracture resistance comparable to conventional fiber post-core restorations followed by full crowns. Importantly, endocrowns were associated with a significantly higher proportion of favorable, restorable failure patterns, suggesting a biomechanical advantage. The absence of intraradicular post placement likely contributes to improved stress distribution and reduced risk of catastrophic root fractures. Clinically, these findings support the use of endocrowns as a conservative and reliable restorative option for endodontically treated posterior teeth with adequate coronal structure. Their use may enhance long-term prognosis while preserving tooth structure and simplifying the restorative procedure.

## Introduction

Endodontically treated teeth often present significant structural compromise due to extensive caries, previous restorations, and loss of tooth structure during access cavity preparation. This reduction in the remaining tooth structure can significantly weaken the tooth and increase its susceptibility to fracture under functional loading [1]. Therefore, appropriate post-endodontic restoration plays a crucial role in restoring the structural integrity and functional longevity of these teeth [1].

Traditionally, endodontically treated posterior teeth with substantial coronal loss have been restored using a post-and-core system, followed by a full-coverage crown. In this approach, an intraradicular post is placed within the root canal to provide retention for the core buildup material, which subsequently supports the final crown restoration [1,2]. Although this method has been widely used and provides satisfactory clinical outcomes, it requires additional removal of radicular dentin for post-space preparation, which may weaken the root and increase the risk of root fracture.

In recent years, endocrowns have emerged as a conservative alternative for restoring endodontically treated posterior teeth, particularly molars with extensive coronal destruction [3]. Endocrowns are monolithic restorations that utilize the pulp chamber for retention instead of relying on intraradicular posts [4]. This design allows the preservation of radicular dentin while providing a large bonding surface for adhesive cementation. By distributing occlusal stresses along the internal walls of the pulp chamber and the remaining tooth structure, endocrowns may enhance fracture resistance and reduce the risk of catastrophic root fractures [5]. In addition to their conservative design and favorable failure patterns, endocrowns offer several clinical advantages, including preservation of pericervical dentin, reduced chairside time, improved adhesive retention, and elimination of complications associated with post placement. They are particularly indicated in cases with extensive coronal destruction, short or curved roots, limited interocclusal space, and when an adequate ferrule cannot be achieved.

With the advancement of adhesive dentistry and computer-aided design and computer-aided manufacturing (CAD-CAM) technologies, endocrowns fabricated from high-strength ceramic materials have gained increasing popularity [6]. Despite their conservative nature and promising clinical outcomes, there remains an ongoing debate regarding their fracture resistance compared to conventional post-and-core restorations followed by full crowns. Therefore, laboratory investigations are necessary to evaluate the mechanical performance of these restorative approaches under controlled conditions.

This in vitro study aimed to compare the fracture resistance of endodontically treated posterior teeth restored with endocrowns and those restored with conventional post-and-core, followed by full crowns. This study aimed to evaluate and compare the fracture resistances of these two restorative approaches in endodontically treated posterior teeth. The objectives were to measure the fracture resistance of teeth restored with endocrowns, assess the fracture resistance of teeth restored with post-and-core followed by a full crown, and compare the fracture resistance between the two restorative techniques to determine the more favorable restorative approach.

## Materials and methods

This in vitro experimental study was conducted in the Department of Conservative Dentistry and Endodontics, Kothiwal Dental College & Research Centre, Moradabad, India, from January 2024 to July 2024. Human mandibular molars of similar size and morphology were selected for the study. Teeth with cracks, caries, restorations, or structural defects were excluded from the study. All selected teeth were cleaned of debris and stored in distilled water at room temperature until use was required. The study was approved by the Institutional Ethical Review Board (KDCRC/IERB/1/2024/SS02).

The sample size was calculated using G*Power software (version 3.1.9, Heinrich Heine University Düsseldorf, Germany). Based on an effect size of 0.6 derived from a previous study [7] comparing the fracture strengths of endodontically treated teeth restored with different post-core systems, the analysis was performed with a power of 80% and a significance level (α) of 0.05. The calculated minimum sample size required was 54 teeth, corresponding to 27 teeth in each group. To compensate for possible specimen loss due to breakage during testing or errors during specimen preparation, an additional 10% of the samples were included. Accordingly, the final sample size was increased to 30 teeth per group, resulting in a total of 60 specimens included in the study.

Standardized endodontic treatment was performed on all teeth. Access cavity preparation was performed using diamond burs (Mani Diamond Burs, Mani Inc., Tochigi, Japan). Root canal instrumentation was completed using rotary nickel-titanium (NiTi) files (ProTaper Gold Rotary File System, Dentsply Sirona, Ballaigues, Switzerland) up to size F2 (25/0.08). The canals were irrigated with 5.25% sodium hypochlorite solution (Parcan Sodium Hypochlorite Solution; Septodont, Saint-Maur-des-Fossés, France), followed by saline irrigation. Obturation was performed using gutta-percha cones (ProTaper Universal Gutta-Percha Points; Dentsply Sirona) and a resin-based sealer (AH Plus Root Canal Sealer; Dentsply Sirona, Konstanz, Germany) using the lateral condensation technique.

Following endodontic treatment, the specimens were randomly divided into two groups of 30 teeth each. Group 1 (endocrown group) (n = 30): Standardized endocrown preparation was performed. The pulp chamber was cleaned and shaped to provide adequate internal retention while maintaining the maximum amount of the remaining tooth structure. Endocrowns were fabricated using lithium disilicate ceramic blocks (IPS e.max CAD Blocks; Ivoclar Vivadent AG, Schaan, Liechtenstein) and CAD-CAM technology. Prior to cementation, the internal surfaces of the restorations were etched with 5% hydrofluoric acid for 20 seconds, rinsed, and air-dried, followed by application of a silane coupling agent and gentle air drying. The tooth surfaces were etched with 37% phosphoric acid for 15 seconds, rinsed, and gently air-dried to maintain moist dentin. A universal adhesive was applied to the tooth surface and light-cured according to the manufacturer’s instructions. Dual-cure resin cement was then applied to the internal surface of the restoration, which was seated under constant finger pressure. Excess cement was removed, and light curing was performed for 20 seconds from all aspects. For the post group, the post space was cleaned, dried with paper points, adhesive was applied, and the fiber post was luted using the same dual-cure resin cement prior to core buildup. Group 2 (post-and-core with full crown group) (n = 30): In this group, post space preparation was performed by removing gutta-percha from the coronal portion of the canal using Gates Glidden drills (Mani Gates Glidden Drills; Mani Inc.) up to size #2. Fiber posts (RelyX Fiber Post; 3M ESPE, St. Paul, Minnesota, USA) were cemented using dual-cure resin cement (RelyX U200 Self-Adhesive Resin Cement, 3M ESPE). Fiber posts of size #2 were used. The core build-up was completed using a composite core material (Filtek Z350 XT Universal Restorative, 3M ESPE). Tooth preparation for full crowns was performed, and crowns were fabricated using lithium disilicate ceramic (IPS e.max CAD; Ivoclar Vivadent AG). The crowns were cemented using dual-cure resin cement.

To ensure standardization and avoid bias, a uniform ferrule height of 1.5-2 mm was maintained in all specimens wherever possible. For the endocrown group, the pulp chamber was standardized to a depth of approximately 3-4 mm to provide adequate internal retention. Additionally, all specimens were prepared to achieve a uniform residual axial wall thickness to minimize variability between samples. To ensure standardization, all teeth selected were mandibular first molars with similar dimensions and free from defects. Root canal treatment was performed by a single operator using a standardized protocol with uniform working length and apical preparation. The remaining coronal tooth structure was standardized before restoration, and all preparations were carried out using calibrated burs under consistent conditions. All restorations were fabricated using the same CAD-CAM system and material, and cementation was performed following a uniform protocol. Specimens were stored under identical conditions prior to testing. During fracture testing, load was applied at a constant crosshead speed and angulation using a universal testing machine to ensure uniform stress application.

All restored specimens were stored in distilled water for 24 hours before mechanical testing. Each specimen was mounted on an acrylic resin block to simulate clinical conditions. Fracture resistance testing was performed using a universal testing machine (Instron Universal Testing Machine, Instron Corporation, Norwood, Massachusetts, USA). A compressive load was applied to the occlusal surface at a crosshead speed of 1 mm/min until fracture. The maximum load at fracture was recorded in newtons (N) for each specimen. The mode of fracture was also observed and classified as favorable (restorable) or unfavorable (non-restorable). The recorded fracture resistance values were compiled and statistically analyzed. 

All statistical analyses were performed using IBM SPSS Statistics for Windows, Version 23.0 (Released 2015; IBM Corp., Armonk, NY, USA). The data distribution was first assessed using the Shapiro-Wilk test to determine whether the fracture resistance values followed a normal distribution. As the data in both groups were normally distributed (p > 0.05), parametric analysis was applied. Descriptive statistics, including the mean, SD, and 95% CIs, were calculated. An intergroup comparison of fracture resistance between the two restorative techniques was performed using an independent samples t-test. The fracture mode frequencies were expressed as percentages. Statistical significance was set at p < 0.05 for all analyses.

## Results

A total of 60 endodontically treated mandibular first molars were included in the study, with 30 specimens restored using lithium disilicate endocrowns (Group 1) and 30 specimens restored using fiber posts, composite cores, and lithium disilicate full crowns (Group 2). Normality testing using the Shapiro-Wilk test confirmed that the fracture resistance values were normally distributed in both groups (Table 1). Group 1 showed a Shapiro-Wilk statistic of 0.965 (p = 0.412), and Group 2 showed a statistic of 0.952 (p = 0.187). As both p-values exceeded 0.05, the assumption of normality was satisfied, justifying the use of parametric tests.

**Table 1:** Tests of normality for fracture resistance values (Shapiro-Wilk test) p > 0.05 denotes no statistical significance.

Group	Shapiro-Wilk statistic	Degree of freedom (df)	p-Value	Distribution
Group 1: Endocrown (lithium disilicate)	0.965	30	0.412	Normal (p > 0.05)
Group 2: Post-core-crown (fiber post + lithium disilicate crown)	0.952	30	0.187	Normal (p > 0.05)

The descriptive statistics for fracture resistance (in Newtons) are presented in Table 2. The mean fracture resistance was 1250.45 ± 152.30 N for the endocrown group (95% CI: 1193.58-1307.32 N) and 1180.20 ± 178.60 N for the post-core-crown group (95% CI: 1113.50-1246.90 N). The mean difference between the groups was 70.25 N, with the endocrown group exhibiting slightly higher fracture resistance. An independent samples t-test revealed no statistically significant difference between the two groups (t = 1.46, p = 0.106).

**Table 2:** Fracture resistance of endodontically treated teeth by restorative technique (in Newtons) p > 0.05 denotes no statistical significance using an independent samples t-test (equal variances assumed).

Group	n	Mean ± SD (N)	95% CI for mean (N)	Mean difference (N)	t-Value	p-Value
Group 1: Endocrown (lithium disilicate)	30	1250.45 ± 152.30	1193.58-1307.32	70.25	1.46	0.106
Group 2: Post-core-crown (fiber post + lithium disilicate crown)	30	1180.20 ± 178.60	1113.50-1246.9			

The failure mode analysis is summarized in Table 3. In the endocrown group, 24 specimens (80%) exhibited favorable (restorable) fracture patterns, including fractures above the cemento-enamel junction (CEJ), debonding, and crown/core fractures without root involvement. Only six specimens (20%) showed unfavorable (non-restorable) fractures, such as vertical root fractures or post/root fractures. In contrast, the post-core-crown group had 12 favorable (40%) and 18 unfavorable (60%) fractures. Overall, while both restorative techniques provided comparable fracture resistance under static loading, endocrowns were associated with a markedly higher proportion of favorable failure modes.

**Table 3:** Failure modes following fracture resistance testing ^††^ Favorable (restorable) fractures: debonding, crown fracture, or fracture above the CEJ without root involvement. ^†††^ Unfavorable (non-restorable) fractures: vertical root fracture, root split, post fracture, or fracture involving the root below the CEJ. Data are presented as frequency (n) and percentage (%). CEJ, cementoenamel junction

Group	n	Favorable (restorable)^††^, n (%)	Unfavorable (non-restorable)^†††^, n (%)
Group 1: Endocrown (lithium disilicate)	30	24 (80%)	6 (20%)
Group 2: Post-core-crown (fiber post + lithium disilicate crown)	30	12 (40%)	18 (60%)

## Discussion

This in vitro study compared the fracture resistance and failure modes of endodontically treated mandibular first molars restored with lithium disilicate endocrowns, fiber posts, composite cores, and lithium disilicate full crowns. The results demonstrated that the endocrowns exhibited slightly higher mean fracture resistance than the post-core-crown group. However, this difference was not statistically significant. More importantly, endocrowns showed a markedly superior failure pattern, with 80% favorable (restorable) fractures compared to 40% in the post-core-crown group.

The comparable fracture resistance between the two groups can be attributed to several biomechanical factors. Both restorations utilized monolithic lithium disilicate ceramics, a material known for its excellent flexural strength (approximately 360-400 MPa) and high fracture toughness [8]. The endocrown design leverages the pulp chamber as a retentive macro-retentive feature while providing a uniform ceramic thickness that effectively distributes occlusal stresses [5]. In contrast, post-core-crown restorations rely on fiber posts for retention, which, although more flexible than metal posts and closer to the dentin modulus, still create areas of stress concentration at the post-dentin interface [2].

Several in vitro studies have reported similar results. Makade et al. [7] observed that different post-core systems varied significantly in fracture resistance, with conventional crowns requiring additional reinforcement. Veselinova et al. [9] and Alzahrani et al. [10] also reported that lithium disilicate endocrowns produced fracture resistance comparable to or slightly higher than traditional crown restorations, attributing this to better stress distribution and adhesive bonding to the remaining tooth structure. Turkistani et al. [11] conducted an in vitro study evaluating the fracture resistance and failure modes of endodontically treated teeth restored with lithium disilicate endocrowns of varying occlusal thicknesses (3 mm, 4.5 mm, and 6 mm). Their findings indicated that the 3 mm thickness group exhibited the highest fracture resistance, supporting the use of thinner endocrowns for improved biomechanical performance while maintaining favorable failure patterns.

The most clinically relevant finding of this study was the significant difference in the failure modes. Endocrowns predominantly failed in a favorable manner (debonding or fracture above the CEJ), making them more amenable to repair than conventional crowns. In contrast, the post-core-crown group showed a higher incidence of unfavorable fractures, including vertical root and post fractures (60%). The higher incidence of favorable failure modes observed in the endocrown group can be attributed to the absence of an intraradicular post and the resulting reduction in stress concentration within the root canal. The monoblock design and adhesive bonding of endocrowns allow more uniform stress distribution along the pulp chamber and remaining tooth structure, leading to failures such as debonding or coronal fractures that are more repairable. In contrast, post-core restorations introduce stress concentration at the post-dentin interface and may create a wedging effect within the root, predisposing the tooth to catastrophic failures such as vertical root fractures, which are typically non-restorable. The advantages of endocrowns are well supported in the literature [3-5]. The absence of an intraradicular post eliminates the wedge effect and reduces the tensile stress within the root canal, thereby lowering the risk of catastrophic root fractures. Systematic reviews have consistently concluded that endocrown restorations exhibit more reparable failure patterns than post-retained restorations [12,13].

From a biomechanical perspective, the endocrown design creates a monoblock effect through adhesive bonding, allowing the restoration and tooth structure to function as a single unit. This improves load transmission and minimizes the dangerous stress concentrations commonly observed in the cervical region in post-core systems [5,12]. Rayyan et al. [14] similarly demonstrated superior fracture strength and predominantly favorable failure modes with endocrowns on compromised mandibular molars compared to post-core restorations without a ferrule. These findings underscore the conservative nature of endocrown preparation, which preserves the pericervical dentin and avoids unnecessary root canal enlargement or post-space preparation risks, such as perforation or strip perforation.

Mezied et al. [15] conducted a comprehensive review comparing endocrowns and post-core retained crowns for restoring root canal-treated molars, highlighting that endocrowns offer a more conservative approach by preserving tooth structure and utilizing the pulp chamber for macro-retention via adhesive bonding. The authors concluded that endocrowns generally performed better than conventional post-core retained crowns in severely compromised molars, with studies showing comparable or superior fracture resistance and success rates (ranging from 94% to 100% in various reports), making them a preferable option when adequate coronal structure remains.

Clinical implications

The findings suggest that lithium disilicate endocrowns are a viable and potentially preferable alternative to conventional post-core-crown restorations for endodontically treated posterior teeth, particularly when adequate coronal structure remains. Endocrowns offer a more conservative, less invasive, and time-efficient treatment option that reduces the risk of non-restorable failures. Clinicians should consider endocrowns in cases with sufficient pulp chamber depth and good remaining walls, as they may improve long-term tooth survival, simplify the restorative protocol, and eliminate the need for crown-lengthening surgery in many situations.

Limitations

This study has certain limitations. Fracture resistance was evaluated using static loading, which does not fully replicate the cyclic fatigue forces encountered in the oral environment. Additionally, the absence of periodontal ligament simulation may have influenced stress distribution within the tooth-restoration complex compared to in vivo conditions. Furthermore, no thermocycling or long-term aging protocol was performed; therefore, the bond strength and fracture resistance values observed may be higher than those expected clinically over time. Future studies should include fatigue testing, different restorative materials, and clinical trials to validate these in vitro results.

## Conclusions

Within the limitations of this in vitro study, lithium disilicate endocrowns demonstrated fracture resistance comparable to that of fiber post-core and lithium disilicate full crown restorations in endodontically treated mandibular first molars, with no statistically significant difference in mean load-to-failure values. However, endocrowns exhibited a markedly higher proportion of favorable failure modes, primarily due to the absence of an intraradicular post and more favorable stress distribution. These findings support the use of endocrowns as a conservative, biomechanically advantageous, and time-efficient restorative option for posterior endodontically treated teeth with adequate remaining coronal structure, potentially improving long-term tooth prognosis and reducing the risk of catastrophic failures.
